# DefectAtlas-20: A real-world image dataset for joint material and surface-condition classification

**DOI:** 10.1016/j.dib.2026.113215

**Published:** 2026-08-27

**Authors:** Ricardo Buettner, Leopold Fischer-Brandies, Lukas Pasold, Luca Eisentraut

**Affiliations:** Chair of Hybrid Intelligence, Helmut-Schmidt-University/University of the Federal Armed Forces Hamburg, Holstenhofweg 85, Hamburg, 22043, Germany

**Keywords:** Surface defects, Visual inspection, Material damage, Scratch detection, Computer vision, Image dataset, Real-world environments, Industrial inspection

## Abstract

Reliable automated surface inspection requires image datasets that reflect the conditions under which material damage occurs in practice. However, many existing defect datasets focus on individual materials, specific components, or controlled imaging environments, limiting their suitability for studying material-dependent surface defect appearance and their performance robustness under real-world variation. This article presents DefectAtlas-20, a dataset of 13,233 RGB photographs organized into 20 classes formed by combining ten commonly encountered material categories with defect and no-defect surface conditions. The materials comprise uncoated and coated metal, uncoated and coated wood, rubber, textile, cardboard, glass, cable, and hard plastic.

Images were acquired using consumer mobile devices in heterogeneous indoor and outdoor field environments around Hamburg, Germany. They capture variation in illumination, viewing angle, background, object geometry, surface texture, reflectance, and usage condition. The defect classes contain naturally occurring damage, while the corresponding no-defect classes show visually comparable surfaces without visible defects. All images were collected according to a predefined protocol, manually labeled, independently reviewed, centrally quality-controlled, anonymized through metadata removal, and converted to lossless PNG format in the sRGB color space.

The released data package includes 512 × 512-pixel images, class and acquisition metadata, and documentation of the collection and curation process. The dataset provides a benchmark for 20-class material-condition classification and additionally supports binary defect recognition, material classification, material-specific defect analysis, transfer learning, and domain-generalization research. Its material diversity and real-world acquisition conditions facilitate the development and evaluation of computer-vision methods for surface inspection, maintenance, refurbishment, and damage assessment.

Specifications Table

**Table:** 

Subject	Computer Sciences
Specific subject area	Computer vision for 20-class material-surface-condition classification of real-world surface defects across common materials.
Type of data	Processed and quality-filtered RGB images (PNG; 512 × 512 px) and an accompanying CSV file containing image-level class labels, image dimensions, and acquisition metadata.
Data collection	Images were captured by multiple contributors with consumer smartphone cameras (makes/models recorded in the metadata) at indoor and outdoor locations. Ten material categories were photographed with and without visible defects under a predefined protocol prohibiting flash, digital zoom, and live-photo modes and specifying distance, angle, focus, lighting, and background. Blurred, poorly exposed, duplicate, mislabeled, or ambiguous images were excluded after contributor and curator review. Accepted files were anonymized, converted to sRGB PNG, and resized to 512 × 512 px.
Data source location	Multiple indoor and outdoor locations in Hamburg and the surrounding metropolitan area, Germany.
Data accessibility	Repository name: DefectAtlas-20: A Real-World Image Dataset for Joint Material and Surface-Condition Classification Data identification number: 10.5281/zenodo.21719370 Direct URL to data: https://doi.org/10.5281/zenodo.21719370
Related research article	none

## 1 Value of the Data

• The dataset provides a **structured benchmark** for **joint material and surface-condition recognition**. Its 20 classes systematically combine ten commonly encountered material categories with **defect and no-defect surface** conditions. The class structure supports **20-class material-condition classification** and can also be decomposed into ten-class material recognition, binary defect recognition, and material-specific defect-classification tasks.
• The images capture visual variation encountered **outside controlled laboratory environments**, including differences in illumination, viewing angle, background, object geometry, surface texture, reflectance, and signs of use. This **diversity** enables researchers to **evaluate model robustness and generalization** across materials, objects, acquisition devices, and environmental conditions relevant to practical visual inspection.
• The no-defect classes contain **visually comparable surfaces** collected from similar materials, object categories, and acquisition contexts as the defect examples. These **contextually related negative samples** create a more challenging classification setting and help reduce reliance on broad differences in object identity, background, or acquisition conditions instead of material-specific surface characteristics.
• The dataset is relevant to researchers and practitioners working on **automated visual inspection, defect recognition, material-aware computer vision, transfer learning, and domain generalization**. It can be used to compare convolutional neural networks, vision transformers, and foundation-model-based approaches and to investigate class imbalance, material-specific error patterns, and cross-material transfer. Potential application domains include **manufacturing inspection, maintenance documentation, refurbishment, resale assessment, and warranty-related surface screening**. The images may also serve as a basis for future extensions with bounding-box or pixel-level defect annotations.

## 2 Background

Automated visual inspection can improve the detection and documentation of surface damage when manual assessment is time-consuming, subjective, or difficult to standardize [1,2]. Image-based defect recognition is relevant to manufacturing quality control, maintenance, refurbishment, warranty assessment, resale grading, and condition monitoring. However, the visual appearance of defects varies substantially with material texture, coating, reflectance, lighting, viewpoint, and object geometry.

Existing datasets have enabled research on concrete cracks [3], road damage [4], pavement cracks [5], pitting on ball screw drive spindles [6], and printed-circuit-board defects [7]. These resources typically focus on a single material, component, defect type, or controlled inspection setting. Smartphone-based datasets, in turn, largely address infrastructure damage rather than defects across heterogeneous everyday materials and objects.

The presented dataset addresses this gap through real-world smartphone images organized into 20 classes that combine ten material categories with defect and no-defect surface conditions. It provides a reusable benchmark for joint material and surface-condition classification under heterogeneous acquisition conditions. The data further support derived tasks such as binary defect recognition, material classification, transfer learning, and domain-generalization studies, and can serve as a basis for future localization or segmentation annotations.

## 3 Data Description

DefectAtlas-20 [8] contains 13,233 RGB images organized into 20 mutually exclusive classes. The classes were formed by combining ten material categories with two surface-condition categories, namely defect and no-defect surfaces. The included materials are uncoated metal, coated metal, uncoated wood, coated wood, rubber, textile, cardboard, glass, cable, and hard plastic. Consequently, each image is assigned to one material-condition class. Table 1 presents the number of images available for each of the 20 classes.

**Table 1:** Distribution of defect and no-defect images across the ten materials.

Material	Number of defect images	Number of no-defect images
Uncoated metal	648	583
Coated metal	607	514
Uncoated wood	830	590
Coated wood	719	632
Rubber	737	626
Textile	645	652
Cardboard	734	692
Glass	666	608
Cable	661	711
Hard plastic	813	565
**Total by condition**	**7,060**	**6,173**
**Total**	**13,233**	

The class taxonomy was defined before image acquisition and was used consistently by all contributors. A defect class contains images in which a visible defect is present on the depicted surface. Depending on the material, defects may appear as grooves, displaced coating, exposed underlying material, changes in surface reflectance, or localized disruptions of the surface texture. The corresponding no-defect class contains surfaces of the same material category without a visible defect. Natural textures, reflections, minor signs of use, and other non-defect irregularities may nevertheless be visible in these images.

The 20-class organization enables models to jointly distinguish the depicted material and its surface condition. This setting is more challenging than binary defect recognition because the visual appearance of defects differs substantially across materials. For example, defects on coated metal may expose the underlying substrate, whereas defects on glass may primarily appear as fine reflective lines. Similarly, material-specific textures can be visually similar to actual surface damage and therefore constitute difficult negative examples.

**Fig. 1:**
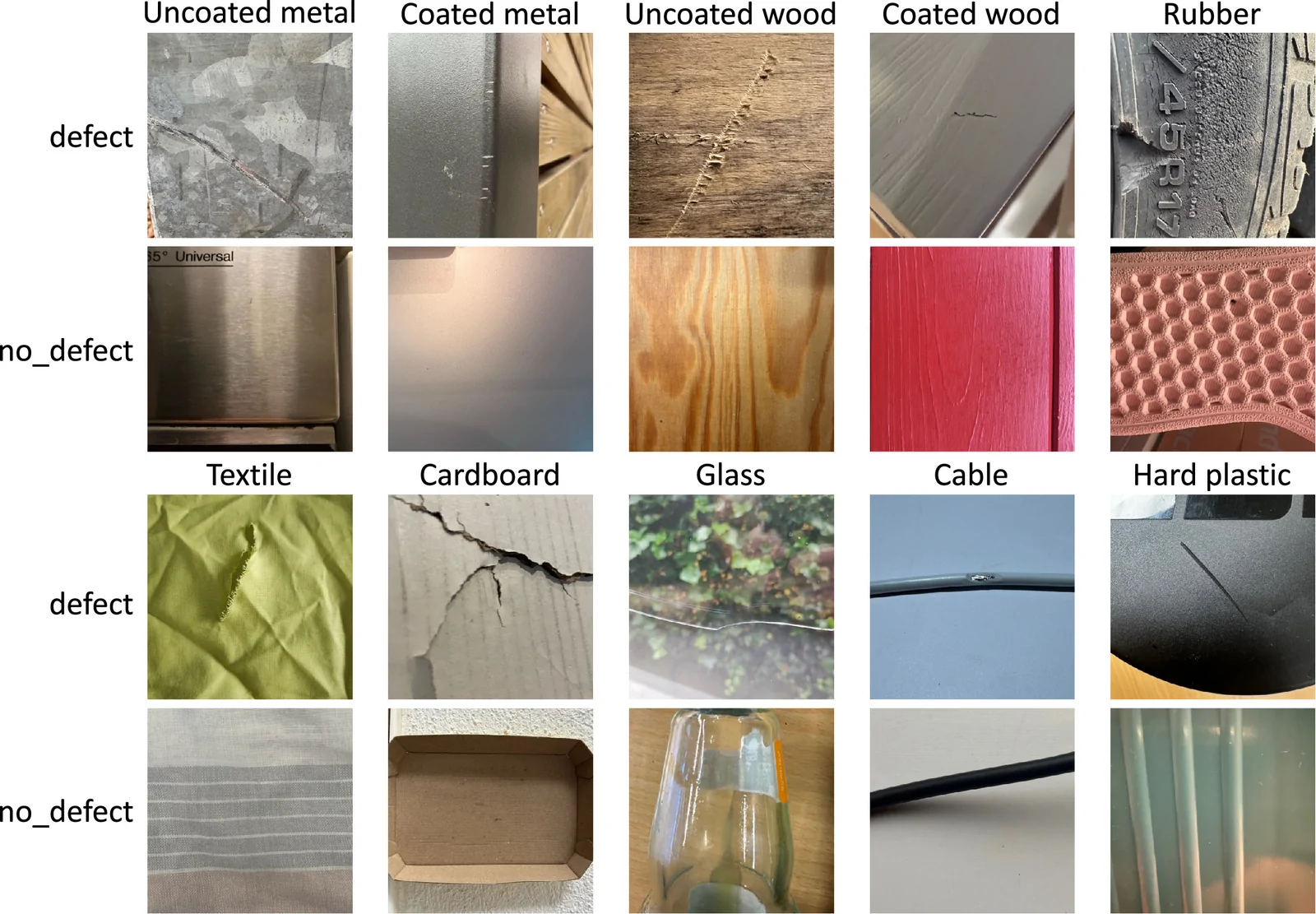
Sample images from the 20 classes formed by combining ten material categories with defect and no-defect surface conditions.

Fig. 1 shows representative images from all 20 classes. The examples illustrate the variation in defect appearance, surface texture, object type, illumination, viewing angle, background, reflectance, and acquisition device. The figure also demonstrates that the no-defect classes contain visually complex surfaces rather than homogeneous or artificially clean reference images.

To quantitatively characterize the visual diversity of the dataset, descriptive image-level statistics were calculated for brightness, saturation, Michelson contrast [9] and Canny edge density [10]. Table 2 summarizes these characteristics across the complete dataset. The observed variation reflects differences in illumination, surface color, texture, reflectance, object geometry, and background complexity under real-world acquisition conditions.

**Table 2:** Descriptive image statistics (mean and standard deviation) for the complete collection.

	Mean	Standard Deviation
**Saturation**	0.2233	0.1622
**Brightness**	0.5112	0.0974
**Michelson Contrast**	0.9296	0.1349
**Canny Edge Density**	0.0243	0.0483

The repository contains two files, images.zip and overview.csv. The images.zip archive contains all 13,233 anonymized RGB images, resized to 512 × 512 pixels and stored as lossless PNG files in the sRGB color space. The overview.csv file provides one image-level record for each released image and contains the corresponding labels, released image dimensions, and available non-identifying acquisition metadata.

Within images.zip, the image files are organized into one directory for each of the 20 material-surface-condition classes. File names follow the pattern [class]_[identifier].png, where the class consists of a two-digit class index, the material category, and either defect or no_defect. The identifier is unique within the corresponding class. For example, 00_glass_defect_00001.png denotes an image assigned to the glass-defect class. The overview.csv file links each image filename to its class, material category, binary surface-condition label, released image dimensions, acquisition device, camera parameters, and other available non-identifying attributes. Table 3 summarizes the columns of overview.csv, including their descriptions, and example values.

**Table 3:** Overview of the columns in overview.csv, including descriptions and representative example records.

Column	Description	Example Record 1	Example Record 2
**sample_name**	File name	04_hard_plastic_ defect_00060.png	19_textile_no_defect _00105.png
**class**	Combined class label	04_hard_plastic_ defect	19_textile_no_defect
**material**	Material category	hard_plastic	textile
**defect_label**	Binary defect status	defect	no_defect
**width**	Image width [px]	512	512
**height**	Image height [px]	512	512
**camera_make**	Camera manufacturer	Apple	Google
**camera_model**	Camera model	iPhone 16	Pixel 7a
**lens_model**	Lens description	iPhone 16 back dual wide camera 2.22mm f/2.2	Pixel 7a back camera 5.43mm f/1.89
**focal_length_mm**	Physical focal length [mm]	2.22	5.43
**focal_length_** **35mm_equiv**	35mm-equivalent focal length [mm]	26.0	25.0
**f_number**	Aperture (f-number)	2.2	1.89
**exposure_time_s**	Exposure time [s]	0.028	0.010005
**iso**	ISO sensitivity	800.0	265.0
**source_format**	Original image format	HEIF	JPEG

In addition to the 20-class classification task, the class labels can be decomposed into a ten-class material label and a binary surface-condition label. This enables secondary experiments on material recognition, binary defect classification, material-specific defect recognition, and cross-material generalization. However, the original directory and annotation structure treats each material-condition combination as a separate class.

## 4 Experimental Design, Materials and Methods

The construction of DefectAtlas-20 followed a two-stage workflow comprising distributed field acquisition and centralized data curation. First, multiple contributors collected images under heterogeneous real-world conditions according to a standardized acquisition protocol. The submitted images were subsequently aggregated, reviewed, processed, and verified through a uniform curation pipeline before their release. This separation was intended to preserve the natural variation introduced by different objects, environments, contributors, and consumer imaging devices while ensuring that labeling, quality control, anonymization through metadata removal, and technical processing were applied consistently across the complete dataset.

### 4.1 Field data collection protocol

Images were captured independently by multiple contributors in Hamburg between October and December 2025 using their own consumer smartphone cameras. To standardize acquisition across different devices while preserving natural real-world variation, contributors were instructed not to use flash, digital zoom, or live-photo capture modes. The smartphone models used for data collection are summarized in Table 4.

**Table 4:** Descriptive statistics and information for the used devices and cameras and the respective number of samples.

Device	Camera Info	Samples
Apple iPhone 16	2.22 mm f/2.2, ∼26 mm eq. (n=1140); 5.96 mm f/1.6, ∼26 mm eq. (n=89)	1,229
Apple iPhone 15 Pro	2.22 mm f/2.2, ∼24 mm eq. (n=962); 6.76 mm f/1.78, ∼24 mm eq. (n=9)	971
Apple iPhone 16 Pro	2.22 mm f/2.2, ∼24 mm eq. (n=908); 6.76 mm f/1.78, ∼24 mm eq. (n=23)	931
Apple iPhone 13	5.1 mm f/1.6, ∼26 mm eq. (n=736); 1.54 mm f/2.4, ∼23 mm eq. (n=19)	755
Apple iPhone 15	5.96 mm f/1.6, ∼26 mm eq. (n=622)	622
Apple iPhone 16 Pro Max	2.22 mm f/2.2, ∼24 mm eq. (n=471); 6.76 mm f/1.78, ∼24 mm eq. (n=2)	473
Samsung SM-G990B	5.4 mm f/1.8, ∼26 mm eq. (n=375)	375
Apple iPhone 15 Plus	5.96 mm f/1.6, ∼26 mm eq. (n=364)	364
Apple iPhone 11	4.25 mm f/1.8, ∼25 mm eq. (n=349)	349
Apple iPhone 13 Pro	5.7 mm f/1.5, ∼26 mm eq. (n=197); 1.57 mm f/1.8, ∼26 mm eq. (n=134); 9.0 mm f/2.8, ∼77 mm eq. (n=2)	333
Apple iPhone 14 Pro	2.22 mm f/2.2, ∼24 mm eq. (n=302); 6.86 mm f/1.78, ∼48 mm eq. (n=10)	312
Apple iPhone 14	5.7 mm f/1.5, ∼26 mm eq. (n=229)	229
Samsung SM-A225F	4.65 mm f/1.8, ∼25 mm eq. (n=217)	217
Samsung SM-S901B	5.4 mm f/1.8, ∼23 mm eq. (n=215)	215
Fairphone FP5	5.56 mm f/1.88 (n=123); 2.19 mm f/2.2 (n=73)	196
Samsung Galaxy S23	5.4 mm f/1.8, ∼23 mm eq. (n=194); 7.0 mm f/2.4, ∼69 mm eq. (n=1)	195
Apple iPhone 11 Pro Max	4.25 mm f/1.8, ∼25 mm eq. (n=195)	195
Samsung Galaxy S24+	unknown lens (n=129); 5.4 mm f/1.8, ∼23 mm eq. (n=66)	195
Apple iPhone 12 mini	4.2 mm f/1.6, ∼26 mm eq. (n=194)	194
Samsung Galaxy A15 5G	3.98 mm f/1.8, ∼26 mm eq. (n=190)	190
Motorola edge 50 pro	5.89 mm f/1.4, ∼25 mm eq. (n=187)	187
HUAWEI MAR-LX1B	4.75 mm f/1.8, ∼27 mm eq. (n=184); 2.7 mm f/2.4, ∼17 mm eq. (n=1)	185
Google Pixel 9a	4.53 mm f/1.7, ∼24 mm eq. (n=182)	182
Samsung Galaxy S24	5.4 mm f/1.8, ∼23 mm eq. (n=177); unknown lens, ∼23 mm eq. (n=3)	180
Apple iPhone 17	5.96 mm f/1.6, ∼26 mm eq. (n=96); 2.22 mm f/2.2, ∼26 mm eq. (n=78)	174
Apple iPhone 12	4.2 mm f/1.6, ∼26 mm eq. (n=161)	161
Apple iPhone 12 Pro	4.2 mm f/1.6, ∼26 mm eq. (n=159)	159
Samsung Galaxy A54 5G	5.54 mm f/1.8, ∼23 mm eq. (n=147)	147
Samsung SM-A336B	4.65 mm f/1.8, ∼25 mm eq. (n=143)	143
Samsung SM-G998B	2.2 mm f/2.2, ∼13 mm eq. (n=135); 6.7 mm f/1.8, ∼24 mm eq. (n=3)	138
Samsung SM-A536B	2.13 mm f/2.4, ∼25 mm eq. (n=84); 5.23 mm f/1.8, ∼24 mm eq. (n=47)	131
Apple iPhone 17 Pro	2.22 mm f/2.2, ∼24 mm eq. (n=129); 6.76 mm f/1.78, ∼24 mm eq. (n=1)	130
Apple iPhone SE (2nd generation)	3.99 mm f/1.8, ∼28 mm eq. (n=124)	124
Google Pixel 7a	5.43 mm f/1.89, ∼25 mm eq. (n=122)	122
Apple iPhone 14 Plus	5.7 mm f/1.5, ∼26 mm eq. (n=122)	122
Apple iPhone 16e	4.2 mm f/1.64, ∼26 mm eq. (n=40)	40
Samsung Galaxy Z Flip7 FE	5.4 mm f/1.8, ∼23 mm eq. (n=6)	6
Apple iPhone 15 Pro Max	2.22 mm f/2.2, ∼24 mm eq. (n=4)	4
Apple iPad	3.3 mm f/2.4, ∼31 mm eq. (n=3)	3
Apple iPad (8th generation)	3.3 mm f/2.4, ∼31 mm eq. (n=1)	1
No EXIF metadata	-	2,354

Contributors positioned the camera as close to the surface as permitted by the device’s minimum focusing distance while keeping the relevant surface region sharply focused and limiting unrelated background content. Devices were held as steadily as possible to reduce motion blur. Strong reflections, specular highlights, pronounced shadows, and visually distracting backgrounds were avoided whenever feasible.

For defect surfaces, contributors were instructed to acquire a corresponding no-defect reference image of the same object or, where this was not possible, of a visually comparable object from the same material category. Reference images were captured under approximately matching viewing distances, angles, illumination conditions, and backgrounds so that the surface condition represented the principal visual difference. All defect examples depicted naturally occurring damage encountered during collection rather than artificially introduced defects.

Before submission, each contributor reviewed their own images and independently examined images collected by another contributor. The review assessed focus, motion blur, exposure, background content, compliance with the prohibited camera settings, material assignment, and surface-condition labeling. Images that did not satisfy the acquisition criteria were replaced or excluded before centralized curation.

### 4.2 Data aggregation

Submitted image files were retained in their device-native formats during transfer and initial aggregation, predominantly JPEG and HEIF/HEIC. Contributors transferred the files directly from their capture devices to a computer using a USB connection or cloud storage. Messaging applications were not used because their automatic recompression could alter the original image content. The dataset curators subsequently pooled the submitted files according to the 20 predefined classes. Each class therefore contains images acquired independently by multiple contributors using different consumer smartphone cameras under the shared acquisition protocol.

Following aggregation, the curators conducted a centralized review independent of the contributor-level quality checks. Each image was manually assessed for compliance with the acquisition protocol, correct material and surface-condition assignment, sufficient defect visibility, and duplicate or near-duplicate content. Images that did not meet these criteria were excluded from the final collection. This centralized review ensured that the same quality and labeling criteria were applied across all contributors, devices, and classes.

Before image conversion, a predefined subset of non-identifying acquisition metadata was extracted from the source files. The retained fields comprise camera make and model, lens model, nominal and 35 mm-equivalent focal length, f-number, exposure time, ISO sensitivity, and source format. These metadata are provided in overview.csv. The source_format column records the format of the original image before conversion to PNG. Individual metadata fields may be unavailable where the corresponding information was not present in the source file.

Because the source images originated from personal smartphones, they could contain metadata unrelated to the depicted material or surface condition, including precise geographic coordinates, capture timestamps, embedded thumbnails, device-specific identifiers, and owner-related information. To remove this information, each image was reconstructed from its decoded pixel data rather than modified in place. Source files were opened using Pillow [11], with the pillow-heif plugin [12] used for HEIF/HEIC files. EXIF orientation information was applied directly to the pixel data before the corresponding tag was discarded [13]. Embedded color profiles, where available, were used to convert the image data to the sRGB color space. The images were subsequently re-encoded so that metadata from the original source files, including GPS coordinates, capture timestamps, embedded thumbnails, software and MakerNote fields, owner-related information, and device serial numbers, were not retained in the released PNG files.

After orientation correction, anonymization, and conversion to the sRGB color space, each image was converted to RGB and resized directly to 512 × 512 pixels using Lanczos resampling. This transformation does not preserve the original aspect ratio. The resulting images were stored as lossless PNG files to avoid introducing additional lossy compression artifacts that could obscure or resemble subtle surface damage. Each accepted image was assigned a class-specific sequential identifier and renamed according to the pattern [class]_[identifier].png. The class component contains the two-digit class index, the material category, and either defect or no_defect. Within images.zip, the released images are organized into one directory for each of the 20 material-surface-condition classes.

### 4.3 Baseline image-classification evaluation

To provide an initial reference benchmark for future reuse of DefectAtlas-20, four established image-classification backbones were trained to assign each image to one of the 20 material-surface-condition classes. The evaluated architectures comprised ResNet-50 [14], Swin Transformer V2 Base [15], EfficientNet-B4 [16], and ShuffleNet V2 [17]. These models represent convolutional and transformer-based architectures with different design principles, computational requirements, and model capacities. Each architecture was initialized with ImageNet-1K pretrained weights, and its original classification layer was replaced with a fully connected output layer containing 20 units.

According to the default image sizes used during pretraining, images were resized to 224 × 224 pixels for the evaluation using ResNet-50 and ShuffleNet V2, to 256 × 256 pixels for the evaluation using Swin Transformer V2 Base and to 380 × 380 pixels for the evaluation using EfficientNet-B4. Model performance was estimated with five-fold stratified cross-validation using shuffled folds and a fixed random seed of 42. Stratification preserved the distribution of all 20 classes across the folds. In each fold, four fifths of the images were used for training and the remaining fifth was used for testing. Training images were augmented by random horizontal flipping, random vertical flipping, and color jittering of brightness, contrast, and saturation. No augmentation was applied to test images.

All model parameters were optimized jointly for a maximum of 50 epochs using the Adam optimizer [18] with an initial learning rate of 1 × 10⁻⁴, weight decay of 1 × 10⁻⁴, and a batch size of 32. Cross entropy loss was used for multiclass classification. A cosine annealing learning rate schedule was applied throughout training [19]. Early stopping was triggered when validation accuracy did not improve for eight consecutive epochs. For each fold, the model state with the highest validation accuracy was retained.

The retained checkpoints were evaluated on their respective held-out test folds. The evaluation metrics were computed according to Eqs. (1)-(4), where TP, TN, FP, and FN denote true positives, true negatives, false positives, and false negatives, respectively.

(1) $$\begin{array}{c} \mathrm{Bal}.\;\mathrm{Accuracy}=\frac{1}{C}\sum_{i=1}^{C}\frac{TP_{i}}{TP_{i}+FN_{i}} \end{array}$$

(2) $$\left(Weighted\right)Precision=\sum_{i=1}^{C}\frac{n_{i}}{N}\times \frac{TP_{i}}{TP_{i}+FP_{i}}$$

(3) $$\left(Weighted\right)Recall=\sum_{i=1}^{C}\frac{n_{i}}{N}\times \frac{TP_{i}}{TP_{i}+FN_{i}}$$

(4) $$F1\;score=\sum_{i=1}^{C}\frac{n_{i}}{N}\times 2\times \frac{Precision_{i}\;\times \;Recall_{i}}{Precision_{i}\;+\;Recall_{i}}$$

C denotes the number of classes, $n_{i}$ the number of samples of class i, and $N=\sum_{i}n_{i}$ the total number of samples. The reported values in Table 5 are the mean and standard deviation across the five test folds for the four evaluated backbones.

**Table 5:** Mean test performance across five folds for each evaluated backbone.

Backbone	Bal. Accuracy	Precision	Recall	F1 score
**ResNet-50 [**14]	66.1 ± 0.9 %	66.9 ± 0.7 %	66.6 ± 1.0 %	66.5 ± 0.9 %
**Swin V2 Base [**15**]**	71.5 ± 1.0 %	72.2 ± 0.8 %	72.0 ± 0.8 %	71.9 ± 0.9 %
**EfficientNet-B4 [**16**]**	71.3 ± 0.9 %	72.1 ± 0.9 %	71.7 ± 0.9 %	71.7 ± 0.9 %
**ShuffleNet V2 [**17**]**	61.9 ± 0.7 %	62.7 ± 0.7 %	62.4 ± 0.8 %	62.2 ± 0.8 %

Across the evaluated backbones, Swin V2 Base achieved the highest mean test balanced accuracy of 71.5% ± 1.0 % across the five folds.

**Fig. 2:**
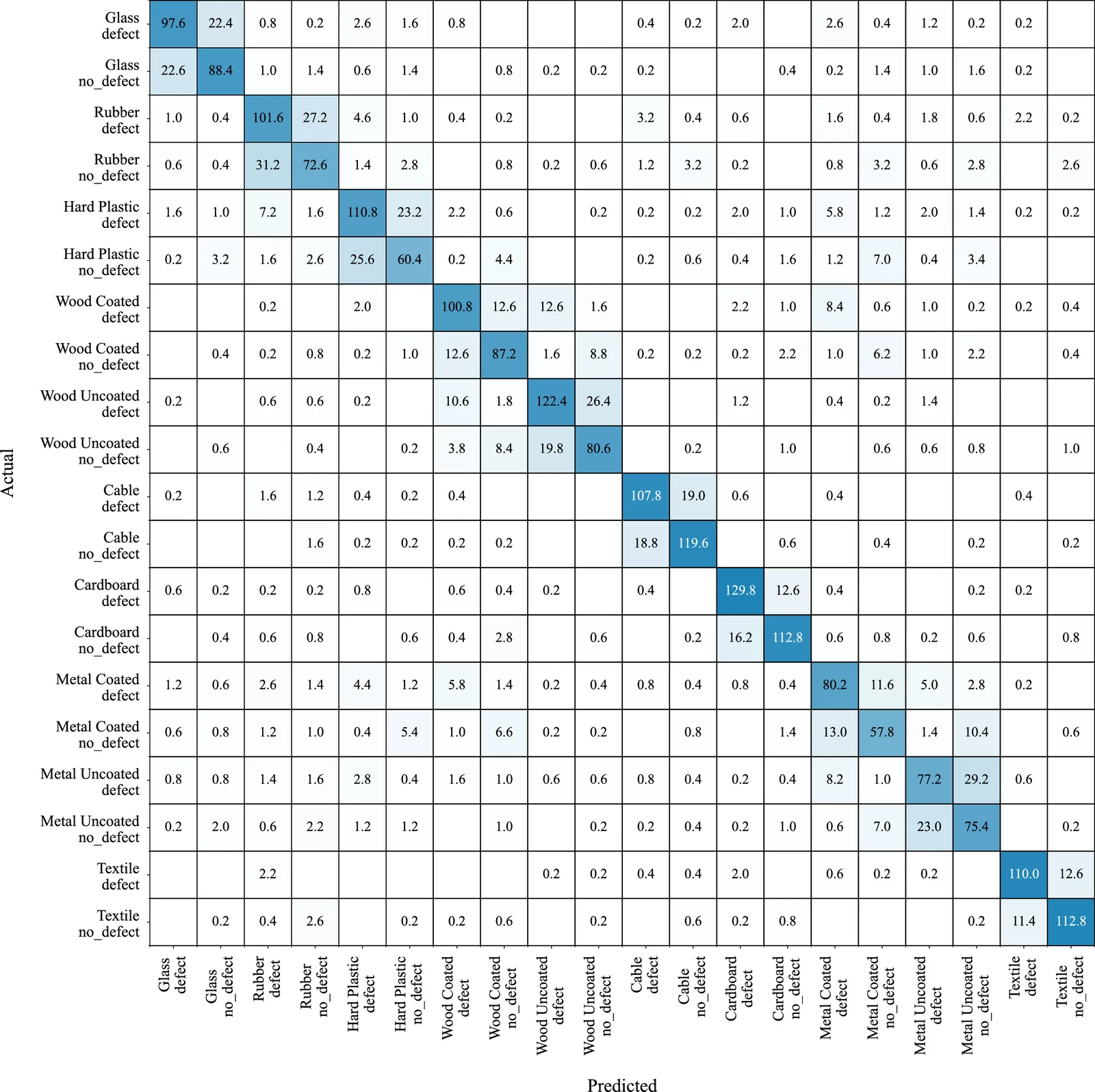
Mean confusion matrix for Swin V2 Base averaged over all five folds.

Fig. 2 presents the 20 × 20 confusion matrix for Swin V2 Base, obtained by averaging the fold-specific confusion matrices across the five cross-validation folds. Cell values denote the mean number of samples per fold. Rows represent the ground-truth material-surface-condition classes, while columns represent the predicted classes. The matrix provides a class-specific view of the classification outcomes and shows whether errors occur predominantly between defect and no-defect variants of the same material or between different material categories.

## Limitations

DefectAtlas-20 was collected by multiple contributors using consumer smartphone cameras in Hamburg and the surrounding metropolitan area between October and December 2025. Although the acquisition protocol intentionally preserves real-world variability, the resulting distribution of devices, environments, objects, and seasonal conditions may introduce collection-specific biases. Model performance may therefore not transfer directly to industrial camera systems, controlled production environments, other geographic regions, or substantially different acquisition conditions.

The dataset provides image-level labels for 20 broad material-surface-condition classes but does not include defect-localization annotations, severity grades, physical defect dimensions, or pixel-level segmentation masks. Naturally occurring defects vary in size, visibility, and appearance, and the defect and no-defect subsets do not constitute a complete one-to-one paired collection. The class distribution is also not perfectly balanced, which should be considered when selecting evaluation metrics and training strategies.

## Ethics Statement

The authors have read and follow the ethical requirements for publication in Data in Brief. The present work does not involve human participants, animal experiments, or data collected from social media platforms.

## Credit Author Statement

**Ricardo Buettner:** Conceptualization, Funding acquisition, Methodology, Project administration, Supervision, Writing - Original Draft, Review & Editing. **Leopold Fischer-Brandies:** Methodology, Validation, Investigation, Data Curation, Writing - Original Draft. **Lukas Pasold:** Validation, Formal analysis, Investigation, Visualization, Writing - Original Draft. **Luca Eisentraut:** Validation, Software, Formal analysis, Investigation, Writing - Original Draft.

## Data Availability

ZenodoDefectAtlas-20: A Real-World Image Dataset for Joint Material and Surface-Condition Classification (Original data)

## References

[1] Nosrati R. (2025). Advancing industrial inspection with an automated visual inspection tool powered by computer vision. Sci. Rep..

[2] Fischer-Brandies L., Bertram S., Mai C., Buettner R. (2025). AsphaltCrackNet: a novel architecture for classifying cracks in asphalt pavement. IEEE Access.

[3] Dorafshan S., Thomas R.J., Maguire M. (2018). SDNET2018: an annotated image dataset for non-contact concrete crack detection using deep convolutional neural networks. Data in Brief.

[4] Arya D., Maeda H., Ghosh S.K., Toshniwal D., Sekimoto Y. (2021). RDD2020: an annotated image dataset for automatic road damage detection using deep learning. Data Brief.

[5] Sabouri M., Sepidbar A. (2023). SUT-Crack: a comprehensive dataset for pavement crack detection across all methods. Data Brief.

[6] Schlagenhauf T., Landwehr M. (2021). Industrial machine tool component surface defect dataset. Data Brief.

[7] Rashid A.J., Ullah M.A., Isfara A., Ahmed N., Mian M., Shalehin M.M. (2025). PCB-defect: an annotated dataset for surface defect detection in printed circuit boards. Data Brief.

[8] Buettner R., Fischer-Brandies L., Pasold L., Eisentraut L. (2026). DefectAtlas-20: a real-world image dataset for joint material and surface-condition classification. Zenodo.

[9] Michelson A.A. (1995).

[10] Canny J. (1986). A computational approach to edge detection. IEEE Trans. Pattern Anal. Mach. Intell. PAMI.

[11] J.A. Clark, et al., Pillow (PIL Fork), version 12.3.0, computer software, 2026. https://python-pillow.github.io/.

[12] A. Piskun, pillow-heif: Python bindings for HEIF images and a plugin for Pillow, version 1.5.0, computer software, 2026. https://github.com/bigcat88/pillow_heif.

[13] P. Harvey, ExifTool, version 13.55, computer software, 2026. https://exiftool.org/.

[14] He K., Zhang X., Ren S., Sun J. (2016). Proceedings of the 2016 IEEE Conference on Computer Vision and Pattern Recognition.

[15] Liu Z., Hu H., Lin Y., Yao Z., Xie Z., Wei Y., Ning J., Cao Y., Zhang Z., Dong L., Wei F., Guo B. (2022). Proceedings of the IEEE/CVF Conference on Computer Vision and Pattern Recognition.

[16] Tan M., Le Q.V. (2019). Proceedings of the 36th International Conference on Machine Learning.

[17] Ma N., Zhang X., Zheng H.-T., Sun J. (2018). Computer Vision - ECCV 2018.

[18] Kingma D.P., Ba J. (2015). in: 3rd International Conference on Learning Representations.

[19] Loshchilov I., Hutter F. (2017). in: 5th International Conference on Learning Representations.

